# Emerging mechanisms by which endocannabinoids and their derivatives modulate bacterial populations within the gut microbiome

**DOI:** 10.3389/adar.2023.11359

**Published:** 2023-12-08

**Authors:** Melissa Ellermann

**Affiliations:** Department of Biological Sciences, University of South Carolina, Columbia, SC, United States

**Keywords:** host microbe interactions, gut microbiome, endocannabinoid, bioactive lipids, bacteria

## Abstract

Bioactive lipids such as endocannabinoids serve as important modulators of host health and disease through their effects on various host functions including central metabolism, gut physiology, and immunity. Furthermore, changes to the gut microbiome caused by external factors such as diet or by disease development have been associated with altered endocannabinoid tone and disease outcomes. These observations suggest the existence of reciprocal relationships between host lipid signaling networks and bacterial populations that reside within the gut. Indeed, endocannabinoids and their congeners such as *N-*acylethanolamides have been recently shown to alter bacterial growth, functions, physiology, and behaviors, therefore introducing putative mechanisms by which these bioactive lipids directly modulate the gut microbiome. Moreover, these potential interactions add another layer of complexity to the regulation of host health and disease pathogenesis that may be mediated by endocannabinoids and their derivatives. This mini review will summarize recent literature that exemplifies how *N-*acylethanolamides and monoacylglycerols including endocannabinoids can impact bacterial populations *in vitro* and within the gut microbiome. We also highlight exciting preclinical studies that have engineered gut bacteria to synthesize host *N-*acylethanolamides or their precursors as potential strategies to treat diseases that are in part driven by aberrant lipid signaling, including obesity and inflammatory bowel diseases.

## Introduction

Host-associated microbial communities and their functional capabilities, collectively referred to as the host microbiome, play integral roles in modulating the health of their hosts and susceptibility to disease. Germ-free experimental models have elegantly demonstrated the dramatic consequences that result from the absence of microbes on host development, metabolism, anatomy, physiology, and behavior. The clear impacts of endogenous microbes on host biology have been further substantiated by gnotobiology, where the introduction of known populations or communities of microbes into germ-free animals promotes defined host responses and health outcomes [1]. Powerful ‘omics approaches such as 16S rRNA sequencing, metagenomics, metatranscriptomics, and metabolomics have been instrumental in correlating specific compositional and functional changes to the host microbiome with certain disease states. These studies have further inspired hypothesis-driven investigations aimed at defining the microbial functions and interactions that contribute to disease pathogenesis. Taken together, studies within the microbiome field have unequivocally demonstrated the importance of these complex and fascinating microbial communities to host biology.

In recent decades, the endocannabinoid system has emerged as an important modulator of gut physiology and homeostasis through its effects on immunity, motility, barrier function, and host metabolism [2]. The endocannabinoid system is comprised of G-protein coupled receptors that are activated by endogenous lipid hormones known as endocannabinoids [3]. The two most well-studied endocannabinoids are 2-arachidonoyl glycerol (2-AG) and arachidonoyl ethanolamide (AEA)—commonly referred to as anandamide [4, 5]. The structure of 2-AG is a monoacylglycerol (MAG) comprised of an arachidonic acid moiety esterified to a glycerol backbone. AEA is an *N-*acyl ethanolamide (NAE) comprised of an arachidonic acid moiety esterified to an ethanolamine backbone. NAEs and MAGs of other acyl lengths and saturation states are also produced by the host including 2-palmitoyl glycerol (2-PG), 2-oleoyl glycerol (2-OG), palmitoyl ethanolamide (PEA), and oleoyl ethanolamide (OEA) [6]. These compounds also act as bioactive lipids that regulate diverse host functions. This mini review will discuss how these two classes of bioactive lipids may also impact the growth and functions of bacteria within the host microbiome, thus expanding the potential effects of these lipids on host physiology.

Endocannabinoid activity is modulated by biosynthetic and degradative enzymes that alter tissue concentrations of these hormones, which function as ligands at cannabinoid receptors through which they exert their physiological effects [7]. Tissue expression profiles of cannabinoids receptors and these biosynthetic and degradative enzymes also impact endocannabinoid tone. Numerous factors have been linked with altered endocannabinoid tone such as diet, stress, and inflammation status [8–12], although the precise molecular mechanisms remain to be elucidated. The gut microbiome is an additional factor that may modulate the endocannabinoid system [13]. Compositional changes to the gut microbiome triggered by dietary interventions or antibiotic treatment correlate with differential expression of endocannabinoid system components and altered profiles of bioactive lipids in the blood stream and in intestinal tissues [14–17]. Moreover, endocannabinoid tone in intestinal tissues is significantly altered in germ free mice compared to conventional mice colonized with a microbiome, suggesting that microbes somehow impact the degradation and/or biosynthesis of NAEs and MAGs [18, 19]. Conversely, pharmacological and genetic interventions that alter host endocannabinoid activity is correlated with an altered gut microbiome [20–27]. Together, these findings suggest that incompletely defined reciprocal relationships exist between the host endocannabinoid system and the gut microbiome. Moreover, the effects of these relationships on host health and susceptibility to disease remain to be fully elucidated.

**FIGURE 1:**
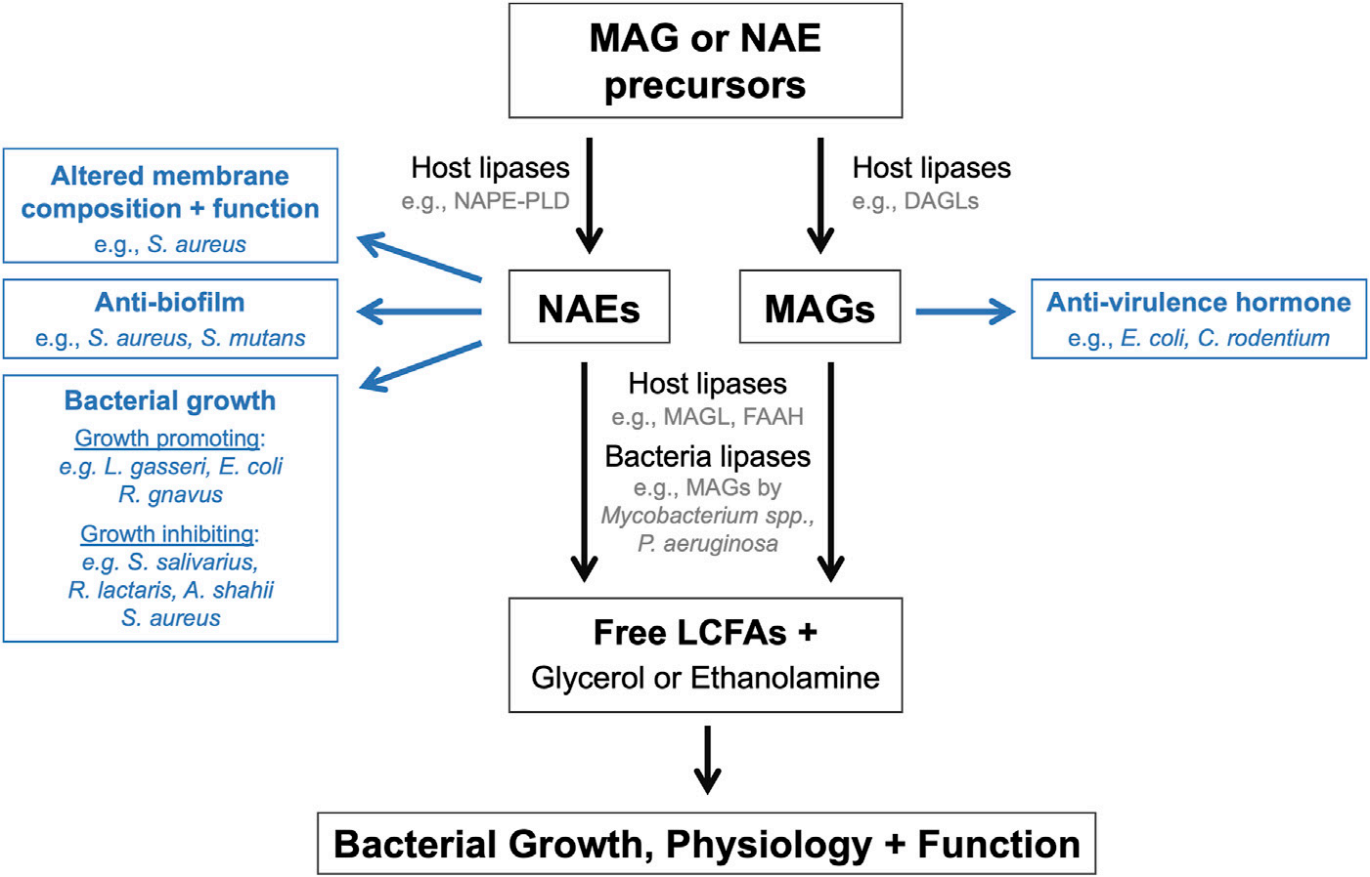
Endocannabinoids and their derivatives directly modulate bacterial populations. Schematic summarizing the mechanisms by which endocannabinoids and their derivatives or breakdown products can influence the growth, physiology, and function of endogenous bacteria within the microbiome.

More recently, experimental evidence has emerged demonstrating that endocannabinoids and their congeners can modulate bacterial functions, physiology, and behaviors (Figure 1). These findings introduce the exciting possibility that these host lipid hormones may directly modulate bacterial populations within host-associated microbial communities such as the gut microbiome. The mini review will summarize literature that exemplifies how endocannabinoids and their derivatives impact bacterial populations *in vitro* and within rodent models. This mini review will also highlight a collection of preclinical studies that have designed genetically engineered bacteria to modulate host NAE levels to treat metabolic and inflammatory diseases. Included research articles were located using the following search terms in the PubMed database: endocannabinoid + bacteria; endocannabinoid + gut microbiome; *N-*acylethanolamide + bacteria; 2-arachidonoyl glycerol + bacteria; anadamide + bacteria. The mechanisms by which the gut microbiome modulates the host endocannabinoid system and the consequent effects on disease development have been reviewed in a companion article for this special issue [24].

## Effects on bacterial growth and metabolism

Numerous studies have correlated compositional changes to the gut microbiome with altered host endocannabinoid tone [20–27]. These ecological changes to the microbial community are likely driven by multiple factors including the indirect effects of host cannabinoid signaling on the gut environment and the direct effects of endocannabinoids on endogenous bacteria. Untargeted metabolomics and metagenomics analyses on fecal samples collected from an IBD patient cohort revealed that NAEs including the endocannabinoid AEA were increased in Crohn’s disease patients relative to ulcerative colitis patients and non-IBD controls [28, 29]. Further experimentation in a murine T-cell transfer colitis model revealed that NAEs are also increased following induction of disease relative to the pre-colitic state [28]. The increased concentrations of luminal NAEs in the inflamed gut corresponded with community-wide changes in the relative abundances of diverse bacterial taxa [28, 29]. These observations prompted the authors to test whether NAEs directly modulate the growth kinetics of gut bacteria [28]. *In vitro* mono-cultures revealed that NAEs—in particular, OEA and linoleoyl ethanolamine (LEA)—enhanced the growth rates and population densities of several bacterial taxa that are elevated in Crohn’s disease patients, including *Escherichia coli, Lactobacillus gasseri*, and *Ruminococcus gnavus* [28]. In contrast, NAEs generally exerted growth inhibitory effects on bacterial taxa depleted in Crohn’s disease patients, including *Streptococcus salivarius, Ruminococcus lactaris,* and *Alistipes shahii* [28]. In a separate collection of studies that sought to investigate the antimicrobial properties of AEA on clinical *Staphylococcus aureus* isolates, AEA exerted bacteriostatic effects on strains grown planktonically and within biofilms [30, 31]. Further analyses via scanning electron microscopy revealed that AEA arrested *S. aureus* replication during late-stage cell division, resulting in larger cells with fully formed septa [30]. Notably, all studies reported strain level variations when evaluating the effects of NAEs on bacterial growth [28, 30–32], therefore suggesting that bacterial strains harbor distinct capabilities in responding to NAEs within their environments.

To evaluate the effects of NAEs on bacterial populations residing within complex microbial communities, Fornelos et al. introduced various combinations of NAEs into an *in vitro* model of the gut microbiota [28], which eliminates any effects of NAEs on the host that may also alter the microbial community. The addition of NAEs to the chemostat cultures altered community composition within 12 h. This was characterized by an increase in several taxa including *Escherichia*, *Enterococcus*, and *Veillonella* species and the depletion of several taxa including *Bacteroides*, *Allistipes*, *Ruminococcus*, and *Clostridium* species. Notably, several of these compositional changes recapitulated putative pathological features of the gut microbiome in Crohn’s disease patients [28, 29, 33, 34]. Together, these findings support the idea that changes in NAE availability within the intestinal lumen during inflammation may promote and/or sustain the ecological changes that drive microbiome dysfunction. To our knowledge, similar studies focused on MAGs have not yet been published. Considering that altered gut endocannabinoid tone and microbiome dysfunction are both associated with numerous disease states including obesity, cardiovascular diseases, metabolic dysfunction, and neurological diseases [14, 24, 35–39], it will be interesting to learn whether the direct effects of NAEs and other endocannabinoid-like molecules on bacterial growth contribute to the pathogenesis of these complex diseases.

The chemical structures of NAEs and MAGs contain potential bacterial nutrients (i.e., long-chain fatty acids—LCFAs, ethanolamine, glycerol) and antimicrobial agents (i.e., LCFAs). This introduces the possibility that NAEs and MAGs may be hydrolyzed into their constituent components to exert their growth inducing or inhibitory effects. In support of this hypothesis, functional bacterial lipases with structural homology to mammalian monoacylglycerol lipases have been reported in environmental bacteria and in *Mycobacterium* species [40–44]. Similarly, *Pseudomonas aeruginosa* encodes an ABHD6-like lipase that can hydrolyze MAGs [45]. Together, these findings demonstrate the potential for bacterial metabolism of endocannabinoids and their congeners within the gut microbiome. In an *in vitro* chemostat model of the gut microbiota, metatranscriptomics analyses revealed that NAEs significantly alter the community transcriptome, which notably included the differential expression of genes involved in LCFA and ethanolamine metabolism [28]. These transcriptional responses suggest that NAEs may be metabolized by certain members within the bacterial community to liberate free LCFAs and ethanolamine. These nutrients may then be consumed by other bacterial taxa, thus exemplifying a putative cooperative interspecific interaction driven by NAE metabolism.

In contrast, transcriptomics studies performed with the gut bacterium *Bacteroides fragilis* cultivated *in vitro* in monocultures revealed that exposure to LEA and AEA induced the upregulation of efflux pumps and the downregulation of the LCFA transporter FadL [28]. Notably, both NAEs inhibited *B. fragilis* growth, suggesting that this bacterium may respond to NAEs by limiting their import and increasing their export to counteract their toxic effects. Notably, free linoleic acid and arachidonic acid can both exert growth inhibitory effects on various bacterial taxa [46, 47]. This introduces the possibility that NAE hydrolysis within the gut microbiome may also be disadvantageous for bacteria that are susceptible to these poly-unsaturated fatty acids.

To summarize, the few studies that have evaluated the effects of endocannabinoids and their congeners on bacterial growth have demonstrated that their effects on microbial ecology are likely complex. Further studies are clearly needed to investigate how NAEs and MAGs impact the growth of bacterial populations within a complex community, both *in vitro* and within a host. Moreover, it will be interesting to investigate how the effects of these bioactive lipids on microbial ecology ultimately modulates host physiology and susceptibility to disease.

## Effects on bacterial physiology and multi-cellular behaviors

The cellular membranes of host-associated bacteria are generally composed of phosphatidylglycerol (PG), phosphatidylethanolamine (PE), and cardiolipin as the major phospholipid species [48]. The fatty acids that are esterified to these phospholipids vary between bacterial strains, but usually range between 14 and 18 carbons and are typically in saturated or mono-unsaturated states. Environmental conditions including stressors that alter membrane function and exogenous lipid availability can modify the relative abundances of specific phospholipids and their fatty acid content within cellular membranes [49, 50]. These structural changes to the membrane can then impact several bacterial functions that including growth, susceptibility to extracellular stressors, and biofilm formation—all of which can subsequently influence host-microbial interactions.

*In vitro* studies performed on bacterial monocultures have demonstrated that host-derived fluids rich in LCFAs—such as bile and serum—impact acyl-LCFA content within bacterial membranes [51–53]. For example, when grown in a nutrient rich medium, the *Enterococcus faecalis* membrane is dominated by vaccenic acid, which comprises approximately 40% of all fatty acid species present [52]. However, when bile is supplemented into this same medium, the percentage of vaccenic acid decreases to about 3%. This corresponds with significant increases in several LCFA species present within the bile including palmitic acid, oleic acid, and stearic acid [52]. These observations suggest that LCFAs within bile are imported by *E. faecalis* and incorporated into phospholipids during membrane biosynthesis. Indeed, when supplied individually, each LCFA dominates fatty acid content within the membrane [52, 54, 55] Similarly, the membrane lipid profile of the nosocomial pathogen *Acinetobacter baumannii* is significantly altered following recovery from pleural lavage fluid in the lungs compared to growth in standard laboratory media [56]. In particular, *A. baumannii* growth within the lungs corresponds with an increase in polyunsaturated LCFA content within membrane PEs. Notably, *de novo* synthesis of polyunsaturated LCFAs was not detected following *in vitro* cultivation in LCFA-free media, suggesting that *A. baumannii* utilizes host-derived PUFAs for membrane biosynthesis during *in vivo* growth. Supporting this hypothesis, genetic inactivation of the main exogenous LCFA transporter FadL conferred a growth defect in *A. baumannii* within several host microenvironments [56]. Notably, studies performed in diverse bacterial taxa—including *Staphylococcus aureus, Escherichia coli, Pseudomonas aeruginosa, Klebsiella pneumonia*, and *Lactobacillus* species—have demonstrated that exogenous LCFA availability influences membrane structure [54, 55, 57–65]. Taken together, these studies clearly demonstrate that LCFAs within host environments can be utilized by bacterial organisms to modify their membrane structures, which in turn may impact host-microbial interactions.

The molecular structure of endocannabinoids and their congeners include an LCFA moiety, and therefore, it is conceivable that these molecules may also impact bacterial membrane physiology and function. In a recent collection of studies, the authors demonstrated that exposure to the endocannabinoid AEA alters lipid content, fluidity, bioenergetics, and permeability of the cytoplasmic membrane in clinical *Staphylococcus aureus* isolates [30–32, 66]. AEA exposure corresponded with increased cardiolipin content within the *S. aureus* membrane [30]. Cardiolipins can form microdomains within bacterial membranes, which in turn can impact the functionality of membrane proteins such as transporters [48]. The addition of AEA to *in vitro* cultures also resulted in decreased efflux of various toxic compounds, including antibiotics, from *S. aureus* cells [30, 31]. These observations corresponded with lower membrane potential, decreased membrane ATPase activity, and increased cardiolipin content, all of which can modulate the functionality of efflux pumps. Decreased efflux associated with AEA also corresponded with the differential expression of various efflux pump genes, which may also contribute to this response [31]. AEA also sensitized clinical *S. aureus* isolates to various antibiotics including beta-lactams, gentamicin, tetracycline, and fluoroquinolones through a mechanism that likely involves compromising efflux pump function [30–32]. The addition of AEA and LEA to *in vitro* cultures also modulated the expression of efflux genes in *B. fragilis* [28]. Together, these studies demonstrate that AEA impacts several compositional and functional aspects of the bacterial cellular membrane. Outstanding questions include whether other NAEs impart similar effects on *S. aureus* growth and membrane function. More broadly, it will be interesting to investigate the effects of NAEs and MAGs on membrane composition and function in other bacterial taxa present within host associated microbial communities and whether these alterations in bacterial physiology impact host disease development.

Within host environments, commensal bacteria and invading pathogens can grow within multicellular structures such as cellular aggregates and biofilms. The formation of these structures involves the biosynthesis and export of components that comprise the eventual extracellular matrix, which serves to adhere bacterial cells together while also acting as a thick protective barrier against environmental insults including the host immune response and antimicrobials. Recent studies have demonstrated that NAEs can exert anti-biofilm effects against *S. aureus* and the oral commensal bacterium *Streptococcus mutans* [30–32, 66, 67]*.* AEA and arachidonoyl serine both synergized with several types of antimicrobial agents to inhibit biofilm formation in clinical *S. aureus* isolates [30–32]. When supplied individually, both compounds inhibited several *S. aureus* behaviors associated with enhanced biofilm formation including surface motility and cell-to-cell aggregation [66]. AEA also altered the gene expression of several biofilm-associated genes and decreased extracellular matrix production in *S. aureus* [31]. Notably, efflux pumps can contribute to biofilm formation by exporting components needed to construct the extracellular matrix. Therefore, it is possible that the inhibitory effects of AEA on efflux pumps as described above may also explain its anti-biofilm effects in *S. aureus*. In *S. mutans,* exposure to either OEA or AEA exacerbated the anti-biofilm effects of the antimicrobial compound poly-L-lysine [67]. In contrast, the NAEs PEA and stearoylethanolamide (SEA) did not impact *S. mutans* biofilm formation, therefore suggesting that this inhibitory effect is unique to NAEs with a monounsaturated LCFA moiety. Taken together, in addition to their effects on membrane physiology, NAEs can also antagonize bacterial behaviors that lead to the formation of biofilms and other multi-cellular structures. Future studies are warranted to investigate how NAEs and other endocannabinoid-like molecules modulate these behaviors within host environments.

## Effects on bacterial signaling

Bioactive lipids function as signaling molecules that are sensed by bacterial organisms to elicit a particular cellular response. Nutrients such as sugars, amino acids, and fatty acids are also sensed by membrane-bound and intracellular receptors that couple nutrient availability with the transcriptional regulation of metabolic pathways and other functions. Bacterial sensing of these environmental cues plays a central role in bacterial pathogenesis and in host-microbial interactions within the gut [68].

A collection of studies over the past decade have demonstrated that free LCFAs act as both nutrients and signals that can modulate a variety of bacterial functions—recently reviewed here [47, 69, 70]. More recently, the endocannabinoid 2-AG was shown to function as a host-derived hormone that is directly sensed by several gut bacterial pathogens including *Citrobacter rodentium* and enterohemorrhagic *E. coli* (EHEC) [71]. *In vitro* functional and biochemical approaches revealed that 2-AG inhibits the membrane-bound bacterial receptor QseC, which functions to stimulate intracellular signaling cascades that activate virulence programs in response to the catecholamines epinephrine and norepinephrine and to the quorum sensing hormone autoinducer-3 [71–75]. In a mouse model of intestinal infection, Magl-deficient mice with elevated levels of 2-AG developed attenuated disease in response to *C. rodentium* challenge [71]. These protective effects were no longer observed when Magl-deficient mice were challenged with *qseC*-deficient *C. rodentium*, suggesting that 2-AG exerts its anti-virulence effects in the gut by inhibiting QseC-dependent virulence. Interestingly, free arachidonic acid—a product released following 2-AG hydrolysis—also exerts anti-virulence effects on EHEC [76]. When imported into EHEC, arachidonic acid is esterified to coenzyme A and then allosterically inhibits the lipid-responsive transcription factor FadR, which in turn represses the expression of virulence genes [76, 77]. Notably, QseC and FadR homologues are present in other bacterial pathogens and commensals [78–81], which introduces the possibility that endocannabinoids and their derivatives may directly modulate the behaviors of many other bacterial organisms.

## Engineering bacteria to modulate host lipid signaling

The first three sections of this mini review summarized experimental evidence that demonstrates how bioactive lipids may directly modulate bacterial populations. Many bacterial taxa within the gut microbiome are currently genetically tractable, therefore introducing the possibility of designing probiotics that specifically target these lipid signaling networks. This last section will summarize two collections of studies that apply this concept to diseases that are in part driven by aberrant NAE signaling—obesity and inflammatory bowel diseases.

Certain NAE species and their *N-*acyl-phosphatidylethanolamine (NAPE) precursors function as satiety signals that are synthesized in the small intestines to regulate host feeding behaviors [82–84]. In rodent models, chronic administration of exogenous NAEs exerts various anorexigenic effects including decreased food consumption and improved host metabolic parameters in rodent models [85]. In one collection of studies, the probiotic *E. coli* strain Nissle was engineered to synthesize and secrete NAEs or their *N-*acyl-phosphatidylethanolamine (NAPE) precursors as a novel therapeutic strategy to treat obesity [86–89]. Chronic administration of NAPE-producing Nissle resulted in decreased weight gain and adiposity in diet-induced and genetic obesity models in a NAPE-PLD dependent manner and in a cardiometabolic disease model [86, 88, 89]. These results corresponded with increased hepatic NAE levels, decreased lipid accumulation and inflammation markers in the liver, and improved metabolic parameters such as glucose tolerance and insulin sensitivity [88]. Similar anti-obesogenic effects were observed with the NAE-producing Nissle strain [89].

In addition to their effects on central host metabolism, certain NAE species such as PEA also exhibit anti-inflammatory properties in experimental colitis models through incompletely defined mechanisms [90–96]. As a strategy to augment local levels of PEA, a second group genetically engineered the probiotic *Lactobacillus paracasei* substrain *Paracasei* F19 to synthesize and secrete PEA when the strain was supplied exogenous palmitic acid [97]. Using a chemically induced model of colitis, the authors demonstrated that co-administration of the PEA-producing *L. paracasei* with palmitate significantly increased intestinal concentrations of PEA and resulted in attenuated colitis development. These protective effects were no longer apparent in mice lacking the PEA receptor PPAR-alpha. In a follow-up study, the authors also demonstrated that this PEA-producing probiotic protected against colitis development induced by the TcdA toxin from *Clostridioides difficile* in a PPAR-alpha dependent manner [98].

Taken together, these studies demonstrate how intestinal probiotic strains can serve as bacterial platforms for delivering NAEs or their precursors to host tissues to treat metabolic and inflammation driven diseases that are characterized by low NAE tone. Because endogenous bacteria within the microbiome likely also synthesize and metabolize NAEs [28, 99], it will be interesting to investigate whether the endogenous microbial production of these bioactive lipids also serve as inputs into host lipid signaling networks, which in turn may also impact the disease development.

## Discussion

This mini review has highlighted the numerous ways in which endocannabinoids and their derivatives directly impact bacterial growth, physiology, and behaviors (Figure 1). These effects have primarily been investigated using *in vitro* single bacterial populations, rather than within polymicrobial communities and/or host environments. Bacterial growth and behaviors are substantially different within host environments and complex microbial community in comparison to *in vitro* conditions. Therefore, future studies are warranted to investigate how these bioactive lipids impact bacteria populations within the gut microbiome in animal models to evaluate whether the effects observe *in vitro* also occur *in vivo*. Approaches to address this question could include microbial sequencing, metabolomics, bacterial and mouse genetics, and gnotobiology. The application of these approaches to established animal models of disease would also begin to address how the regulation of bacterial growth and behaviors by these bioactive lipids may impact disease pathogenesis and susceptibility. Finally, while not the focus of this mini review, it is important to acknowledge that endocannabinoid activity also impacts the function of host cell populations within the intestines, which in turn can modulate gut physiology and microbiome function. However, it remains unclear how the distinct effects of endocannabinoid activity on host tissues and microbial populations, and on the reciprocal interactions between host and microbe, together ultimately impact the establishment and maintenance of gut homeostasis and the development of disease. This represents an additional exciting avenue of research highly worth exploring.
